# Validity of the international physical activity questionnaire and the Singapore prospective study program physical activity questionnaire in a multiethnic urban Asian population

**DOI:** 10.1186/1471-2288-11-141

**Published:** 2011-10-13

**Authors:** Ei Ei Khaing Nang, Susan Ayuko Gitau Ngunjiri, Yi Wu, Agus Salim, E Shyong Tai, Jeannette Lee, Rob M Van Dam

**Affiliations:** 1 Department of Epidemiology and Public Health, Yong Loo Lin School of Medicine, National University of Singapore, Singapore, Republic of Singapore; 2 Department of Medicine, Yong Loo Lin School of Medicine, National University of Singapore, Singapore, Republic of Singapore; 3 Department of Nutrition, Harvard School of Public Health, Boston MA, USA

## Abstract

**Background:**

Physical activity patterns of a population remain mostly assessed by the questionnaires. However, few physical activity questionnaires have been validated in Asian populations. We previously utilized a combination of different questionnaires to assess leisure time, transportation, occupational and household physical activity in the Singapore Prospective Study Program (SP2). The International Physical Activity Questionnaire (IPAQ) has been developed for a similar purpose. In this study, we compared estimates from these two questionnaires with an objective measure of physical activity in a multi-ethnic Asian population.

**Methods:**

Physical activity was measured in 152 Chinese, Malay and Asian Indian adults using an accelerometer over five consecutive days, including a weekend. Participants completed both the physical activity questionnaire in SP2 (SP2PAQ) and IPAQ long form. 43subjects underwent a second set of measurements on average 6 months later to assess reproducibility of the questionnaires and the accelerometer measurements. Spearman correlations were used to evaluate validity and reproducibility and correlations for validity were corrected for within-person variation of accelerometer measurements. Agreement between the questionnaires and the accelerometer measurements was also evaluated using Bland Altman plots.

**Results:**

The corrected correlation with accelerometer estimates of energy expenditure from physical activity was better for the SP2PAQ (vigorous activity: r = 0.73; moderate activity: r = 0.27) than for the IPAQ (vigorous activity: r = 0.31; moderate activity: r = 0.15). For moderate activity, the corrected correlation between SP2PAQ and the accelerometer was higher for Chinese (r = 0.38) and Malays (r = 0.57) than for Indians (r = -0.09). Both questionnaires overestimated energy expenditure from physical activity to a greater extent at higher levels of physical activity than at lower levels of physical activity. The reproducibility for moderate activity (accelerometer: r = 0.68; IPAQ: r = 0.58; SP2PAQ: r = 0.55) and vigorous activity (accelerometer: 0.52; IPAQ: r = 0.38; SP2PAQ: r = 0.75) was moderate to high for all instruments.

**Conclusion:**

The agreement between IPAQ and accelerometer measurements of energy expenditure from physical activity was poor in our Asian study population. The SP2PAQ showed good validity and reproducibility for vigorous activity, but performed less well for moderate activity particularly in Indians. Further effort is needed to develop questionnaires that better capture moderate activity in Asian populations.

## Background

Globally, non communicable diseases (NCDs), consisting mainly of cardiovascular diseases, cancers, chronic respiratory diseases and diabetes make up to 60% of all deaths [1]. WHO projects that NCD deaths will increase by 17% over the next ten years with the highest absolute number of deaths occurring in Asia [1]. Physical inactivity has been identified as a modifiable shared risk factor for NCDs [1]. Although the health benefits of physical activity in preventing cardiovascular diseases, type 2 diabetes, several cancers, and even poor mental health has been well established, the level of physical activity has been declining in many countries [2]. This may be due to several factors including increased reduced occupational and household activity due to mechanization and reduced transport activity due to replacement of walking and cycling by transport using -cars, trains and buses. Leisure time activity may have increased due to greater popularity of sports activities or decreased due to more time spend on TV, computer games and the internet. However in order to describe, monitor and possibly implement effective interventions for physical activity it is important to measure activity levels accurately and across multiple domains of physical activity (transportation, leisure-time, occupational and household) within the population studied.

There are different methods for assessing physical activity. These include criterion methods such as doubly labeled water, indirect calorimetry, and direct observation; objective methods such as heart rate monitor, pedometer, accelerometer; and subjective methods including questionnaires and activity diaries [3-6]. However, the instrument used in large scale epidemiological studies has generally been the questionnaire because of low cost, ease of administration and relative ease of calculating energy expenditure [7].

The International Physical Activity Questionnaire (IPAQ) was designed to provide a set of well-developed instruments that can be used internationally to obtain estimates of physical activity that can be compared across different populations [8]. In order to interpret the findings from these questionnaire-based studies, it is important that the questionnaire is validated against objective assessments in the population of interest. The IPAQ has been validated in multiple populations, but within Asia only the Japanese and Hong Kong Chinese population have been studied [8-10]. In addition, the Japanese validation study only evaluated total physical activity and not the ability of IPAQ to differentiate between moderate and vigorous activity [8]. The two validation studies conducted in Hong Kong Chinese reported inconsistent results [9,10]. Thus, we would like to assess the measurement properties of IPAQ separately for moderate and vigorous activity in Singapore, a developed urban multi-ethnic Asian country.

Between 2003 and 2007 we conducted a population-based study, the Singapore Prospective Study Program (SP2) [11], which collected data on physical activity. The SP2 Physical Activity questionnaire (SP2PAQ) was adapted from several established questionnaires developed in Western nations [12-14] to assess transportation, occupation, leisure time and household activities. The ability of the SP2PAQ and the IPAQ, which assesses similar domains of physical activity, to assess physical activity in Asian populations has not been evaluated. This is important because the validity of other physical activity questionnaires used in Asian population such as the questionnaire in the Shanghai physical activity study had limited validity for moderate-to-vigorous intensity (spearman correlation of 0.17)[15].

The aim of this study was to assess the validity of IPAQ long form in a multi-ethnic population of Chinese, Malays, and Indians living in Singapore and compares it against the SP2PAQ using accelerometer measurements as the reference instrument.

## Methods

### Study population

We studied 164 participants, aged above 21 years. These were mainly students and staff from local university and hospital. Ethics approval was obtained from the National University of Singapore Institutional Review Board (NUS IRB). Written informed consent was obtained from all participants. One participant withdrew from the study after 2 days. Hence, 163 participants completed the study.

### Procedures

Anthropometric measurements were taken (height to the nearest 0.1 cm and weight to the nearest kg). Participants then completed one of the two evaluated questionnaires (SP2PAQ or IPAQ) before the physical activity monitoring period. Physical activity was monitored using an Actical accelerometer for five consecutive days including 3 weekdays and 2 weekend days. Participants were instructed to wear the accelerometer for all waking hours except during water-based activities. After completion of the five- day monitoring period, participants returned the accelerometer and completed the remaining questionnaire. The first 120 participants answered IPAQ before the monitoring period and SP2PAQ immediately after the monitoring period. The order of the questionnaires was reversed for the last 43 participants to evaluate whether the order of questionnaire assessment may have affected the results.

Of the 163 participants, 52 participants were re-recruited to test reproducibility of the questionnaires and the accelerometer. The reproducibility of the accelerometer was evaluated at the same time as the questionnaires were administered using the same device in both periods. The mean interval between the two assessments was 175 days (SD = 64 days) with minimum 63 days and maximum 308 days.

### Physical activity questionnaires

The 8 versions of International physical activity questionnaire (IPAQ) were developed by an International Consensus Group between 1997 and 1998. These were developed as an instrument for cross-national monitoring of physical activity with a recall period of 7 days. In 2000, the reliability and validity of the questionnaires were evaluated in 12 countries and the result was published in 2003, which showed acceptable reliability and validity [8,16]. Since its development, it has been validated in different populations and also widely used in research studies [10,17-19]. The self-administered IPAQ long form covers four domains of physical activity: job related activity; transportation activity; housework, house maintenance, and caring for family; and recreation, sports, and leisure-time physical activity. For each domain, the time spent on moderate and vigorous activity per day and the numbers of days per week were recorded. Walking time was asked in all domains except household activity. In addition, time spent sitting on weekday and weekend was also recorded.

The physical activity questionnaire used in Singapore Prospective Study Program (SP2PAQ) is an interviewer-administered questionnaire with a recall period of the previous 3 months. As mentioned before it was adapted from several established questionnaires validated in other populations [12-14] and encompassed transportation, occupation, leisure time and household activities.

The questions on transportation activity were adapted from National Health Survey 2004 questionnaire [20] which asked about walking or cycling for transport for at least 10 minutes. The duration, frequency and the intensity of the activity (light, moderate, or vigorous) were recorded. Questions on occupational activity were based on the validated Modifiable Activity Questionnaire [13,21]. Participants were asked to list all jobs held during the past 3 months. For each job entry, data was collected for the job schedule and job activity was determined by the number of hours spent sitting at work and the most common physical activities performed when not sitting. Leisure time activity was adapted from the Minnesota leisure time activity questionnaire covering a total of 48 specific activities and open questions about possible other activities which has been validated in various populations [14,22-24]. For each activity, participants identified the frequency and the average duration of participation in each activity. Household activity was adapted from the Yale physical activity questionnaire which covers housework, yard work and caretaking for elderly persons or children and has been validated in diverse populations [12,25,26]. Participants were asked about the type of activity performed and the frequency and duration of each activity. The SP2PAQ can be found in additional file 1.

### Actical physical activity monitor

Objective measurement of physical activity was obtained by using the Actical^® ^physical activity monitor (Mini Mitter Co., Inc., Bend, OR) which is a water resistant, lightweight (17 g) and small (28 × 27 × 10 mm) device. The monitors are initialized and downloaded through the ActiReader PC serial port interface. According to manufacturer, the Actical is an omnidirectional, piezoelectric accelerometer, which is able to detect movements in all directions. It is sensitive to movements in the range of 0.5-3 Hz and its sensitivity allows for detection of sedentary movement as well as high-energy movements. Its reduced frequency range also minimizes the effect of undesirable noise impulses, which tend to skew energy expenditure [27]. The Actical accelerometer has been validated previously showing good reliability and accuracy for estimating the energy expenditure from physical activity and the time spent in moderate and vigorous physical activity [28,29] and has been used in epidemiological studies [30]. The physical activity intensity prediction of the Actical accelerometer was validated with a room calorimeter. This showed that differences between the measurements of the Actical accelerometer and the calorimeter for the time spent in each moderate and vigorous intensity activity was < 2%[31].

Compared to ankle and wrist, hip was the best location for monitor placement to predict the energy expenditure from physical activity when validated against with VmaxST portable metabolic system (R = 0.90)[32]. For this study, all participants wore the Actical accelerometer on the right hip, just anterior to iliac crest with elastic belt. The device was initialized using 15-s epochs and converted to 1-min epochs for data analysis of energy expenditure.

The quality of the devices was monitored by checking the coefficient of variation (CV) of the devices monthly [33]. All the devices were placed on the mechanical shaker for 12 hours and the CV was calculated by dividing the standard deviation of activity counts with the mean of activity counts captured by the devices. The CVs during the study period were acceptable, ranging from 10.2% to 16.6%.

### Calculation of Energy Expenditure from Physical Activity

For IPAQ, we used the IPAQ data processing rules [34] for our calculations. The data was truncated at 21 hours per week for each of the following groups of activities: walking activity, other moderate activity, and vigorous [34]. Subsequently, walking activity and other moderate activity were combined to derive total moderate activity. Metabolic equivalent task (MET) levels were obtained from the IPAQ scoring protocol [34] for the IPAQ questionnaire and from the compendium by Ainsworth et al [35] for the SP2PAQ questionnaire. One MET unit is defined as the energy expenditure for sitting quietly, which for the average adult is approximately 3.5 ml of oxygen × kg bodyweight^-1 ^× min^-1 ^or 1 kcal × kg body weight^-1 ^× h^-1 ^[35]. For both questionnaires, minutes were converted to hours and weekly energy expenditure from each physical activity (Kcal/week) was calculated as follows: hours spent on activity per day × numbers of days per week × MET value × body weight in kg [36,37]. Then the energy expenditures from all the activities under each intensity category were combined to obtain the total energy expenditure per week for each moderate and vigorous intensity category. Moderate intensity was defined as 3 to 6 METs and vigorous intensity was defined as more than 6 METs [38].

The resulting measures from the two questionnaires expressed in Kcal per week were divided by 7 and the total Kcal for each moderate and vigorous intensity category from accelerometer for 5-day wearing period was divided by 5, to derive average Kcal per day for all methods.

The Actical accelerometer recorded physical activity in a series of activity counts which were proportional to the magnitude and duration of the sensed accelerations. The raw minute-by-minute activity counts were then transformed into energy expenditure by the computer program using MET prediction algorithms of Klippel et al [32]. The output of the program included data of energy expenditure (Kcal/day) and time spent on light, moderate and vigorous activity with cutoff points of 3 METs between light and moderate activity and 6 METs between moderate and vigorous activity. In this study, we used 2R regression to estimate energy expenditure from physical activity, which exhibits a decreased tendency to over predict energy expenditure [27].

### Statistical Analysis

The accelerometer data was considered valid only when 10 or more hours of data per day were collected for five days. Thus, 8 participants were excluded because they did not meet this criterion. In addition, 3 participants who reported the sum total of all walking, moderate and vigorous time more than 16 hours per day in IPAQ were treated as outliers and excluded from analysis according to IPAQ data processing rule [34]. As a result, 152 participants were included in the analysis and 43 participants for reproducibility analysis. The correlations between estimates of energy expenditure from physical activity assessed by the accelerometer and estimates assessed by questionnaires were obtained by the Spearman rank correlation test. Because correlations between the questionnaires and the reference instrument (i.e. the accelerometer) are interpreted as measures of the accuracy of the questionnaires, it is desirable to correct for limitations of the reference instrument that reduce these correlations. Thus we calculated correlation coefficients corrected for within-person variation in the accelerometer measurements using the formula suggested by Beaton et al [39].

$${\text{r}}_{\text{t}}={\text{r}}_{0}\sqrt{1+\frac{\lambda \text{accelerometer}}{\text{naccelerometer}}}$$

where

r_t _= "true" correlation coefficient

r_0 _= observed correlation coefficient

$$\lambda \text{accelerometer}=\frac{1-\text{ICCaccelerometer}}{\text{ICCaccelerometer}}$$

where ICC_accelerometer _= interclass correlation coefficient of accelerometer

n_accelerometer _= number of repeated accelerometer measurements within-subject

The 95% and 99.99% confidence intervals for correction correlations were calculated using the formulas suggested by Willett et al [40]. In addition, a Bland-Altman plot was created for the agreement between the questionnaires and the accelerometer measurement. The reliability of the questionnaires was evaluated using Spearman rank correlation coefficients. All statistical analyses were performed using Stata 10 for Windows (Stata Corporation, College station, Texas, USA).

## Results

The study population (N = 152) had mean age of 38.3 years and mean BMI of 24.54 kg/m^2^. The majority of participants had a job, but there were also students, homemakers, retired and unemployed participants. Nearly 70% had a higher education while 21% achieved secondary education and less than 10% had no education or primary level. There was a large variation in household income among participants (Table 1).

**Table 1:** Socio-demographic characteristics of study population

N = 152	
Age(years), mean ± SD	38.30 ± 12.86
Body Mass Index(kg/m^2^), mean ± SD	24.54 ± 4.64
	
**Age group (N, %)**	
≤ 40 years	87(57.24)
> 40 years	65(42.76)
	
**Gender (N, %)**	
Male	64(42.11)
Female	88(57.89)
	
**Ethnicity (N, %)**	
Chinese	66(43.42)
Malay	34(22.37)
Indian	52(34.21)
	
**Highest level of education (N, %)**	
None/primary	13(8.55)
Secondary	32(21.05)
Technical school/diploma	42(27.63)
University	65(42.76)
	
**Work status (N, %)**	
Working	98(64.47)
Student	33(21.71)
Homemaker	17(11.18)
Retired	3(1.97)
Unemployed	1(0.66)
	
**Household income(S$/month) (N, %)**	
Less than $2000	27(17.76)
$2000 to $3999	40(26.32)
$4000 to $5999	37(24.34)
$6000 to $9999	27(17.76)
More than $ 10 000	19(12.5)
Decline to answer	2(1.32)

Additional file 2 shows the correlation between the IPAQ and SP2PAQ. The two questionnaires showed reasonable correlation with each other for moderate activity (r = 0.55), but a low correlation for vigorous activity (r = 0.27). Table 2 presents data on the Spearman rank correlation between energy expenditure from physical activity assessed using questionnaires and the accelerometer. In general, correlations were higher for vigorous activity than moderate activity and higher for the SP2PAQ than for the IPAQ. The correlations between the IPAQ and accelerometer were 0.13 for moderate activity, 0.18 for vigorous activity, and 0.19 for moderate and vigorous activity combined. These correlations remained low after correction for within-person variation in the accelerometer measurements; the corrected correlation was 0.15 for moderate activity was and 0.31 for vigorous activity. The correlation for the SP2PAQ was 0.24 for moderate activity, 0.42 for vigorous activity, and 0.28 for moderate and vigorous activity combined. Correction of these correlation coefficients for within-person variation in the accelerometer measurements increased the correlation only slightly for moderate activity (r = 0.27), but substantially for vigorous activity (r = 0.73). No substantial difference in correlation between the questionnaires and accelerometer was observed according to the order of the questionnaire assessments (i.e. before or after the accelerometer assessment) (data not shown).

**Table 2:** Correlation between the IPAQ and SP2PAQ measurements and accelerometer measurements of energy expenditure from physical activity.

	Moderate activity		Vigorous activity		
N = 152	Correlation	Corrected Correlation¥	Correlation	Corrected Correlation¥	
**IPAQ**	0.13	0.15	0.18*	0.31*	
Stratified by age group					
≤40 years (N = 87)	0.08	0.09	0.30*	0.52*	
> 40 years (N = 65)	0.21	0.24	-0.07	-0.01	
Stratified by gender					
Male (N = 64)	0.24	0.27	0.28*	0.48	
Female (N = 88)	0.12	0.13	0.05	0.09	
Stratified by ethnicity					
Chinese (N = 66)	0.32*	0.36*	0.20	0.35	
Malay (N = 34)	0.19	0.21	0.07	0.12	
Indian (N = 52)	-0.15	-0.17	0.28*	0.48	
**SP2PAQ**	0.24*	0.27*	0.42**	0.73*	
Stratified by age group					
≤40 years (N = 87)	0.21	0.24*	0.48**	0.83	
> 40 years (N = 65)	0.27*	0.30*	0.28*	0.48	
Stratified by gender					
Male (N = 64)	0.16	0.18	0.49**	0.85	
Female (N = 88)	0.29*	0.33*	0.34*	0.59*	
Stratified by ethnicity					
Chinese (N = 66)	0.34*	0.38*	0.50**	0.87	
Malay (N = 34)	0.51*	0.57	0.39*	0.68	
Indian (N = 52)	-0.08	-0.09	0.32*	0.55	

*p value < 0.05, ** p value < 0.0001

IPAQ = International Physical Activity Questionnaire; SP2PAQ = Singapore Prospective Study Program Physical Activity Questionnaire

¥ corrected correlation = corrected for within-person variation in accelerometer measurements.

The validity of the questionnaires was further assessed by stratifying the study population according to age, gender, and ethnic group. Compared with the younger age group, the correlation between the energy expenditure from physical activity assessed by questionnaire and accelerometer in the older group tended to be higher for moderate activity, but lower for vigorous activity. This was observed for both questionnaires. The correlation was higher in men for both moderate and vigorous activity when the IPAQ was used, whereas the correlation was higher in women than men for moderate activity when the SP2PAQ was used.

The performance of the SP2PAQ was similar in all three ethnic groups for vigorous activity, but for moderate activity, Malays showed a higher correlation with accelerometer measurements than Chinese and particularly Indians. For the IPAQ, reasonable correlations were only observed with the accelerometer in Chinese for moderate activity and Indians for vigorous activity.

**Figure 1:**
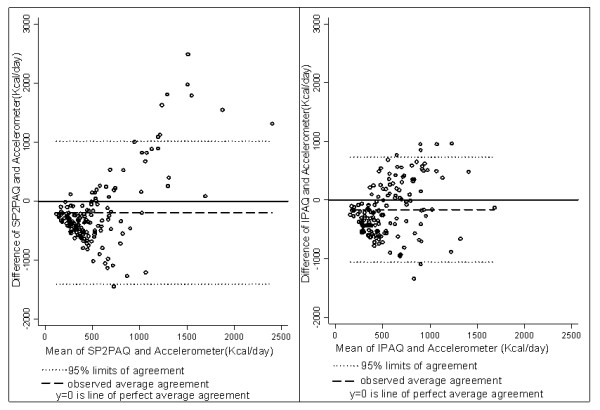
**Bland-Altman plot for comparing the agreement between questionnaires and accelerometer measurements of moderate activity**. The difference of estimate of moderate physical activity from the questionnaire and the accelerometer (y-axis) are depicted in relation to the mean of estimates of moderate physical activity from the questionnaire and the accelerometer (x-axis).

**Figure 2:**
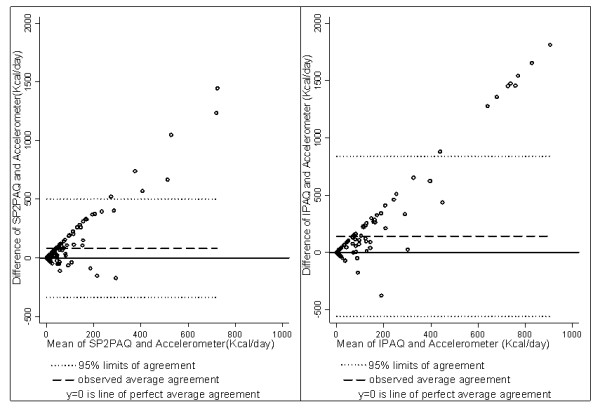
**Bland-Altman plot for comparing the agreement between questionnaires and accelerometer measurements of vigorous activity**. The difference of estimate of vigorous physical activity from the questionnaire and the accelerometer (y-axis) are depicted in relation to the mean of estimates of vigorous physical activity from the questionnaire and the accelerometer (x-axis).

The agreement between the questionnaires and the accelerometer was also evaluated using Bland-Altman plots. Both IPAQ and SP2PAQ underestimated average energy expenditure from moderate activity, but overestimated average energy expenditure from vigorous activity as compared with the accelerometer. The mean difference of daily energy expenditure between the measurements of IPAQ and accelerometer for moderate activity was -169 Kcal/day (95%CI: -236 to -90) and that of between SP2PAQ and accelerometer was -196 Kcal/day (95% CI: -295 to -97). SP2PAQ showed good agreement with the accelerometer for moderate activity when the energy expenditure was below approximately 1200 Kcal per day. However, it tended to overestimate energy expenditure when energy expenditure increased above that level (Figure 1). For vigorous activity, the mean difference of daily energy expenditure between the measurements of the IPAQ and accelerometer was 139 Kcal per day (95% CI: 82 to 196) and that of between SP2PAQ and accelerometer was 81 Kcal per day (95% CI: 47 to 116). For vigorous activity, both questionnaires showed good agreement with the accelerometer for energy expenditure below approximately 400 Kcal per day. However, the higher the energy expenditure above that level, the greater was the degree of overestimation of the questionnaires (Figure 2).

The reproducibility of the two questionnaires and the accelerometer was also evaluated (Table 3). IPAQ had higher reproducibility for moderate activity, but lower reproducibility for vigorous activity than the SP2PAQ. The reproducibility of the accelerometer was higher than the two questionnaires for moderate activity, but lower than SP2PAQ for vigorous activity.

**Table 3:** Reproducibility of IPAQ, SP2PAQ, and accelerometer measurements of energy expenditure from physical activity.

N = 43	IPAQ		SP2 PAQ		Accelerometer	
	**Moderate activity**	**Vigorous activity**	**Moderate activity**	**Vigorous activity**	**Moderate activity**	**Vigorous activity**

Spearman correlation	0.58**	0.38*	0.55**	0.75**	0.68**	0.52*

*p value < 0.05, ** p value < 0.0001

IPAQ = International Physical Activity Questionnaire; SP2PAQ = Singapore Prospective Study Program Physical Activity Questionnaire

## Discussion

In our study in a developed multi-ethnic urban Asian population, SP2PAQ showed a substantially higher correlation with an objective measure of energy expenditure from physical activity than the IPAQ for both moderate and vigorous activity. The validity of the IPAQ for ranking the physical activity level of individuals was inadequate in our population, whereas the validity for SP2PAQ was acceptable for this purpose with the possible exception of ranking moderate activity in Indians. Both questionnaires tended to overestimate energy expenditure for vigorous activity, especially at higher levels of energy expenditure. For moderate activity, both questionnaires underestimated the energy expenditure when compared with the measurement of accelerometer. The reproducibility over an average of 6 months of the two questionnaires and the accelerometer was reasonably good.

Our study showed that the corrected correlation for vigorous activity was substantially better than for moderate activity, and this is consistent with findings in other studies [41,42]. In the Stanford Five-City Project, a survey of a representative population sample of four cities in central California, which compared nine measurement instruments for physical activity, recall was accurate for vigorous activity, but poor for moderate activity and this finding was consistent in men and women and in all domains of activity [41]. The same finding was observed in the validation of the Stanford 7-Day Recall, which showed lower correlation for moderate activity than vigorous activity in men (0.23 vs. 0.59) [42].

The correlation between IPAQ and accelerometer in our population appears to be lower than other populations [18,43]. In the New Zealand population the correlation was 0.19 for moderate activity and 0.42 for vigorous activity [43] and in a Swedish population it was 0.21 for moderate activity and 0.71 for vigorous activity [18]. This may be due to differences in culture as well as educational level of participants that might affect interpretation of the questionnaire [8]. All participants in the Swedish study had a higher education level, whereas our study population consisted of participants with varying educational levels. However, when compared with other validation studies in Asia, the correlation between IPAQ and accelerometer for the Chinese ethnic group in our study was similar to the Chinese population studied in Hong Kong (r = 0.27 for moderate activity and r = 0.28 for vigorous activity) [10]. Another validation study in the Chinese population of Hong Kong showed different results according to the accelerometer used: the correlations of IPAQ with the Tritrac accelerometer were 0.15 and 0.18 for moderate and vigorous activity respectively, whereas with the MTI accelerometer these correlations were -0.06 and 0.44 respectively. In a Japanese population, the correlation between the IPAQ and accelerometer measurements of total physical activity was 0.36 [8].

The validity of SP2PAQ is comparable to other questionnaires that have been used in large epidemiological studies. For example the correlation of Behavioural Risk Factor Surveillance System (BRFSS) physical activity questionnaire used for monitoring physical activity across the U.S.A. compared with the accelerometer was 0.31 for moderate activity and 0.17-0.26 for vigorous activity [44], whereas the correlation of New Zealand Physical Activity Questionnaire(NZPAQ-LF) with accelerometer was 0.30 for moderate activity and 0.37 for vigorous activity [43].

When we compared questionnaire and accelerometer estimates of energy expenditure from physical activity using Bland-Altman plots, greater differences between the two methods were observed with increasing means of measurements for both moderate and vigorous activity. This may be due to either the questionnaire increasingly over-estimating activity with increasing activity or the accelerometer increasingly under-estimating activity with increasing activity. In a study done by Klesges et al reported that participants overestimated the duration of their physical activities, especially for aerobic activities [45]. In addition, the Actical accelerometer may have underestimated energy expenditure, especially at high levels of energy expenditure [46]. The accelerometer is known to substantially underestimate energy expenditure for specific activities [47]. For example, accelerometers have limitations in detecting activities where the body is mostly stationary such as when cycling or weight lifting [47]. Moreover, in our study, the accelerometer was taken off during water-based activities. This may have reduced the amount of activity detected by the accelerometer as compared with the questionnaire although only five participants reported swimming during the period in which they wore the accelerometer. The combination of over-estimation by the questionnaires and under-estimation by the accelerometer may have given rise to the observation that the difference between these methods was greater at higher levels of activity. Similar findings were reported for a nationally representative sample of the Swedish population, where the difference between the IPAQ and accelerometer measurements of time spent on physical activity was larger at higher activity levels reported by the IPAQ [48].

Several methodological differences exist between SP2PAQ and IPAQ. It should be noted that the IPAQ assesses physical activity in the past week, whereas the SP2PAQ assesses habitual physical activity of at least the past 3 months. For the first 120 participants, the week recorded by IPAQ was different from that of the week measured by the accelerometer as IPAQ was administered before the accelerometer wearing period. However, we reversed the order of questionnaire administration for the subsequent 43 participants so that the IPAQ questionnaire applied to the same week of accelerometer measurement and found that the order of questionnaire administration did not affect the agreement with accelerometer measurements. The mode of administration of the questionnaires was also different as SP2PAQ is administered by an interviewer whereas IPAQ is self-administered. In a comprehensive review of physical activity instruments, it was concluded that the accuracy of interviewer-administered questionnaires tends to be greater than for self-administered questionnaires [49]. Finally it should be noted that the primary intention of IPAQ is to obtain comparable population estimates of physical activity data across different countries, whereas the aim of SP2PAQ was to assess inter individual variation in usual physical activity within a population.

To our knowledge, this is one of a few studies that validated physical activity questionnaires in an Asian population. In addition, the correlations were corrected for within-person variation in the accelerometer measurements. Within-person correlation in the reference instrument will reduce the correlation with the evaluated questionnaires and should be corrected for in validation studies [50]. The drop-out rate in our study was negligible as there was only one person who withdrew from the study. There are also several limitations in our study that need to be considered. The size of our study population was modest and most participants were from a hospital and a university campus thus limiting generalizability. However, the participants were derived from fairly wide age and socioeconomic groups with different educational, occupational and income levels. Although the distributions of age, gender and ethnicity were not exactly the same across the sub-groups, these differences in distribution were not statistically significant. The reference measurement used in this study was the accelerometer which is not the gold standard to validate the physical activity measurements [51]. However, the current reference standard for validating activity questionnaires, the doubly labeled water technique, is not only very costly but it also does not provide information on the patterns of physical activity as it estimates total energy expenditure [51]. The accelerometer on the other hand can provide the frequency, duration and intensity of free living physical activity to obtain a good estimate of energy expenditure and has been recommended as an objective method of choice to use in validating questionnaires or studying patterns of physical activity [6,52,53]. It has also been used to validate physical activity questionnaires in national surveys such as the England Physical Activity questionnaire [54] and the BRFSS [44]. Finally, the interval between the test and retest measurements was rather long. We realize that as a result the reliability estimates are affected by both measurement error related to the assessment of short-term activity and real changes in activity habits of participants over time. However, in epidemiological studies we are generally interested in habitual activity over years as this is most relevant for the development of chronic diseases. For this application, an inability of assessment methods to capture long-term physical activity is therefore a limitation and long-term reproducibility, part of which may be due to real changes in physical activity, is most relevant.

## Conclusion

Our study showed that the IPAQ had limited accuracy for distinguishing physical activity levels of individuals and performed poorly in our study population as compared with Western populations. However, its performance was comparable to that observed in a Chinese population in Hong Kong. The SP2PAQ had acceptable validity and reproducibility and can be used in large epidemiological studies particularly for the assessment of vigorous physical activity. For moderate activity, however, adaptation of the questionnaire for the Indian population may be needed. For both the IPAQ and SP2PAQ questionnaire, considerable measurement error observed for the estimation of absolute physical activity levels particularly at higher levels of activity, which should be taken into account when the adequacy of activity levels of population groups are assessed. When we compare our results for IPAQ with results from studies in other populations, it is evident that the validity of the IPAQ differs substantially between populations. Thus, validation sub-studies using objective measures of physical activity within large epidemiological studies are desirable to quantify measurement error and later correct estimates of physical activity and health outcomes for measurement error.

## Competing interests

The authors declare that they have no competing interests.

## Authors' contributions

EEKN was responsible for data collection, statistical analysis and drafting the manuscript. SAGN and YW involved in data collection and helped with the data analysis. AS helped in data analysis. EST and JL directed the study and helped in revising the manuscript. RMVD helped in data analysis, interpretation of the results and led writing of the manuscript. All authors read and approved the final manuscript.

## Pre-publication history

The pre-publication history for this paper can be accessed here:

http://www.biomedcentral.com/1471-2288/11/141/prepub

## Supplementary Material

Additional file 1 **"Singapore Prospective Study Program Physical Activity Questionnaire (SP2PAQ)" for the questionnaire used to assess physical activity in Singapore Prospective Study Program**. Click here for file

Additional file 2 **"Correlation between IPAQ and SP2PAQ measurements of energy expenditure from physical activity" for spearman correlations between IPAQ and SP2PAQ measurements of energy expenditure from moderate and vigorous activity**. Click here for file

## References

[B1] World Health Organisation.2008-2013 Action plan for the global strategy for the prevention and control of noncommunicable diseases http://whqlibdoc.who.int/publications/2009/9789241597418_eng.pdf

[B2] World Health Organisation.Global Recommendations on Physical activity for Health 2010 http://whqlibdoc.who.int/publications/2010/9789241599979_eng.pdf 26180873

[B3] Westerterp KR Assessment of physical activity level in relation to obesity: current evidence and research issues Med Sci Sports Exerc 1999 31 11 Suppl S522 525 10593522 10.1097/00005768-199911001-00006

[B4] Schutz Y Weinsier RL Hunter GR Assessment of free-living physical activity in humans: an overview of currently available and proposed new measures Obes Res 2001 9 6 368 379 10.1038/oby.2001.48 11399784

[B5] Lamonte MJ Ainsworth BE Quantifying energy expenditure and physical activity in the context of dose response Med Sci Sports Exerc 2001 33 6 Suppl S370 378 discussion S419-320 11427762 10.1097/00005768-200106001-00006

[B6] Bassett DR Jr Validity and reliability issues in objective monitoring of physical activity Res Q Exerc Sport 2000 71 2 Suppl S30 36 10925822

[B7] Paffenbarger RS Blair SN Lee IM Hyde RT Measurement of physical activity to assess health effects in free-living populations Med Sci Sports Exerc 1993 25 1 60 70 10.1249/00005768-199301000-00010 8423758

[B8] Craig CL Marshall AL Sjostrom M Bauman AE Booth ML Ainsworth BE Pratt M Ekelund U Yngve A Sallis JF International physical activity questionnaire: 12-country reliability and validity Med Sci Sports Exerc 2003 35 8 1381 1395 10.1249/01.MSS.0000078924.61453.FB 12900694

[B9] Macfarlane DJ Lee CC Ho EY Chan KL Chan D Convergent validity of six methods to assess physical activity in daily life J Appl Physiol 2006 101 5 1328 1334 10.1152/japplphysiol.00336.2006 16825525

[B10] Macfarlane D Chan A Cerin E Examining the validity and reliability of the Chinese version of the International Physical Activity Questionnaire, long form (IPAQ-LC) Public Health Nutr 2010 1 8 10.1017/S1368980010002806 20939939

[B11] Nang EE Khoo CM Tai ES Lim SC Tavintharan S Wong TY Heng D Lee J Is there a clear threshold for fasting plasma glucose that differentiates between those with and without neuropathy and chronic kidney disease?: the Singapore Prospective Study Program Am J Epidemiol 2009 169 12 1454 1462 10.1093/aje/kwp076 19406920

[B12] Dipietro L Caspersen CJ Ostfeld AM Nadel ER A survey for assessing physical activity among older adults Med Sci Sports Exerc 1993 25 5 628 642 8492692

[B13] Kriska AM Knowler WC LaPorte RE Drash AL Wing RR Blair SN Bennett PH Kuller LH Development of questionnaire to examine relationship of physical activity and diabetes in Pima Indians Diabetes Care 1990 13 4 401 411 10.2337/diacare.13.4.401 2318100

[B14] Taylor HL Jacobs DR Jr Schucker B Knudsen J Leon AS Debacker G A questionnaire for the assessment of leisure time physical activities J Chronic Dis 1978 31 12 741 755 10.1016/0021-9681(78)90058-9 748370

[B15] Peters TM Shu XO Moore SC Xiang YB Yang G Ekelund U Liu DK Tan YT Ji BT Schatzkin AS Validity of a physical activity questionnaire in Shanghai Med Sci Sports Exerc 2010 42 12 2222 2230 10.1249/MSS.0b013e3181e1fcd5 20404770 PMC2975050

[B16] Bassett DR Jr International physical activity questionnaire: 12-country reliability and validity Med Sci Sports Exerc 2003 35 8 1396 10.1249/01.MSS.0000078923.96621.1D 12900695

[B17] Kurtze N Rangul V Hustvedt BE Reliability and validity of the international physical activity questionnaire in the Nord-Trondelag health study (HUNT) population of men BMC Med Res Methodol 2008 8 63 10.1186/1471-2288-8-63 18844976 PMC2577099

[B18] Hagstromer M Oja P Sjostrom M The International Physical Activity Questionnaire (IPAQ): a study of concurrent and construct validity Public Health Nutr 2006 9 6 755 762 16925881 10.1079/phn2005898

[B19] Rutten A Ziemainz H Schena F Stahl T Stiggelbout M Auweele YV Vuillemin A Welshman J Using different physical activity measurements in eight European countries. Results of the European Physical Activity Surveillance System (EUPASS) time series survey Public Health Nutr 2003 6 4 371 376 12795825 10.1079/PHN2002450

[B20] Ministry of Health, Singapore:National Health Survey 2004 http://www.moh.gov.sg/content/moh_web/home/Publications/Reports/2005/national_health_survey_2004.html

[B21] Schulz LO Harper IT Smith CJ Kriska AM Ravussin E Energy intake and physical activity in Pima Indians: comparison with energy expenditure measured by doubly-labeled water Obes Res 1994 2 6 541 548 16355515 10.1002/j.1550-8528.1994.tb00103.x

[B22] Elosua R Marrugat J Molina L Pons S Pujol E Validation of the Minnesota Leisure Time Physical Activity Questionnaire in Spanish men. The MARATHOM Investigators Am J Epidemiol 1994 139 12 1197 1209 8209878 10.1093/oxfordjournals.aje.a116966

[B23] Richardson MT Leon AS Jacobs DR Jr Ainsworth BE Serfass R Comprehensive evaluation of the Minnesota Leisure Time Physical Activity Questionnaire J Clin Epidemiol 1994 47 3 271 281 10.1016/0895-4356(94)90008-6 8138837

[B24] Starling RD Matthews DE Ades PA Poehlman ET Assessment of physical activity in older individuals: a doubly labeled water study J Appl Physiol 1999 86 6 2090 2096 10368377 10.1152/jappl.1999.86.6.2090

[B25] Kolbe-Alexander TL Lambert EV Harkins JB Ekelund U Comparison of two methods of measuring physical activity in South African older adults J Aging Phys Act 2006 14 1 98 114 16648654 10.1123/japa.14.1.98

[B26] Moore DS Ellis R Allen PD Cherry KE Monroe PA O'Neil CE Wood RH Construct validation of physical activity surveys in culturally diverse older adults: a comparison of four commonly used questionnaires Res Q Exerc Sport 2008 79 1 42 50 18431950 10.1080/02701367.2008.10599459

[B27] Heil DP Predicting activity energy expenditure using the Actical activity monitor Res Q Exerc Sport 2006 77 1 64 80 16646354 10.1080/02701367.2006.10599333

[B28] Esliger DW Tremblay MS Technical reliability assessment of three accelerometer models in a mechanical setup Med Sci Sports Exerc 2006 38 12 2173 2181 10.1249/01.mss.0000239394.55461.08 17146326

[B29] Rothney MP Schaefer EV Neumann MM Choi L Chen KY Validity of physical activity intensity predictions by ActiGraph, Actical, and RT3 accelerometers Obesity (Silver Spring) 2008 16 8 1946 1952 10.1038/oby.2008.279 PMC2700550 18535553

[B30] Tremblay M Wolfson M Gorber SC Canadian Health Measures Survey: rationale, background and overview Health Rep 2007 18 Suppl 7 20 18210866

[B31] Rothney MP Schaefer EV Neumann MM Choi L Chen KY Validity of physical activity intensity predictions by ActiGraph, Actical, and RT3 accelerometers Obesity 2008 16 8 1946 1952 10.1038/oby.2008.279 18535553 PMC2700550

[B32] Klippel NJ Heil DP Validation of Energy Expenditure Prediction Algorithms in Adults Using the Actical Electronic Activity Monitor Medicine & Science in Sports & Exercise 2003 35 5 S284

[B33] Ward DS Evenson KR Vaughn A Rodgers AB Troiano RP Accelerometer use in physical activity: best practices and research recommendations Med Sci Sports Exerc 2005 37 11 Suppl S582 588 16294121 10.1249/01.mss.0000185292.71933.91

[B34] International Physical Activity Questionnaire. Guidelines for Data Processing and Analysis of the International Physical Activity Questionnaire (IPAQ)-Short and Long Forms http://www.ipaq.ki.se/scoring.pdf

[B35] Ainsworth BE Haskell WL Whitt MC Irwin ML Swartz AM Strath SJ O'Brien WL Bassett DR Jr Schmitz KH Emplaincourt PO Compendium of physical activities: an update of activity codes and MET intensities Med Sci Sports Exerc 2000 32 9 Suppl S498 504 10993420 10.1097/00005768-200009001-00009

[B36] The compendium of physical activities http://purl.access.gpo.gov/GPO/LPS53360

[B37] Ransdell LB Wells CL Physical activity in urban white, African-American, and Mexican-American women Med Sci Sports Exerc 1998 30 11 1608 1615 10.1097/00005768-199811000-00009 9813874

[B38] Physical Activity Guidelines Advisory Committee.Physical Activity Guidelines Advisory Committee Report, 2008 2008 Washington, DC: U.S.Department of Health and Human Services

[B39] Beaton GH Milner J Corey P McGuire V Cousins M Stewart E de Ramos M Hewitt D Grambsch PV Kassim N Sources of variance in 24-hour dietary recall data: implications for nutrition study design and interpretation Am J Clin Nutr 1979 32 12 2546 2559 506977 10.1093/ajcn/32.12.2546

[B40] Rosner B Willett WC Interval estimates for correlation coefficients corrected for within-person variation: implications for study design and hypothesis testing American journal of epidemiology 1988 127 2 377 386 3337089 10.1093/oxfordjournals.aje.a114811

[B41] Sallis JF Haskell WL Wood PD Fortmann SP Rogers T Blair SN Paffenbarger RS Jr Physical activity assessment methodology in the Five-City Project Am J Epidemiol 1985 121 1 91 106 3964995 10.1093/oxfordjournals.aje.a113987

[B42] Richardson MT Ainsworth BE Jacobs DR Leon AS Validation of the Stanford 7-day recall to assess habitual physical activity Ann Epidemiol 2001 11 2 145 153 10.1016/S1047-2797(00)00190-3 11164131

[B43] Boon RM Hamlin MJ Steel GD Ross JJ Validation of the New Zealand Physical Activity Questionnaire (NZPAQ-LF) and the International Physical Activity Questionnaire (IPAQ-LF) with accelerometry Br J Sports Med 2010 44 10 741 746 10.1136/bjsm.2008.052167 18981036

[B44] Yore MM Ham SA Ainsworth BE Kruger J Reis JP Kohl HW Macera CA Reliability and validity of the instrument used in BRFSS to assess physical activity Med Sci Sports Exerc 2007 39 8 1267 1274 10.1249/mss.0b013e3180618bbe 17762359

[B45] Klesges RC Eck LH Mellon MW Fulliton W Somes GW Hanson CL The accuracy of self-reports of physical activity Medicine and science in sports and exercise 1990 22 5 690 697 10.1249/00005768-199010000-00022 2233209

[B46] Crouter SE Churilla JR Bassett DR Jr Estimating energy expenditure using accelerometers European journal of applied physiology 2006 98 6 601 612 10.1007/s00421-006-0307-5 17058102

[B47] Troiano RP Translating accelerometer counts into energy expenditure: advancing the quest Journal of Applied Physiology 2006 100 4 1107 1108 16540708 10.1152/japplphysiol.01577.2005

[B48] Hagstromer M Ainsworth BE Oja P Sjostrom M Comparison of a subjective and an objective measure of physical activity in a population sample Journal of physical activity & health 2010 7 4 541 550 20683097 10.1123/jpah.7.4.541

[B49] Sallis JF Saelens BE Assessment of physical activity by self-report: status, limitations, and future directions Res Q Exerc Sport 2000 71 2 Suppl S1 14 10925819

[B50] Rimm EB Giovannucci EL Stampfer MJ Colditz GA Litin LB Willett WC Reproducibility and validity of an expanded self-administered semiquantitative food frequency questionnaire among male health professionals Am J Epidemiol 1992 135 10 1114 1126 discussion 1127-1136 1632423 10.1093/oxfordjournals.aje.a116211

[B51] Melanson EL Jr Freedson PS Physical activity assessment: a review of methods Crit Rev Food Sci Nutr 1996 36 5 385 396 10.1080/10408399609527732 8725670

[B52] Freedson PS Miller K Objective monitoring of physical activity using motion sensors and heart rate Res Q Exerc Sport 2000 71 2 Suppl S21 29 10925821

[B53] Westerterp KR Plasqui G Physical activity and human energy expenditure Curr Opin Clin Nutr Metab Care 2004 7 6 607 613 10.1097/00075197-200411000-00004 15534427

[B54] Basterfield L Adamson AJ Frary JK Parkinson KN Pearce MS Reilly JJ Longitudinal study of physical activity and sedentary behavior in children Pediatrics 2011 127 1 e24 30 10.1542/peds.2010-1935 21173005

