# Supplementary material for: Validity of the international physical activity questionnaire and the Singapore prospective study program physical activity questionnaire in a multiethnic urban Asian population
Source: BMC Med Res Methodol. 2011 Oct 13;11:141. doi: 10.1186/1471-2288-11-141 (PMC3212806; doi:10.1186/1471-2288-11-141)
Supplement: Additional file 1 — "Singapore Prospective Study Program Physical Activity Questionnaire (SP2PAQ)" for the questionnaire used to assess physical activity in Singapore Prospective Study Program. [file 1471-2288-11-141-S1.DOC]

**Additional file 1.Singapore Prospective Study Program Physical Activity questionnaire (SP2PAQ)**

**The following questionnaire will be used to assess the amount of physical activity you do. All questions relate to the last 3 months.**

**When we ask about the intensity of physical activity, the following categories will apply:**

**1. Transportation**

**1.1** Do you walk for at least 10 minutes continuously to get to and from places?

Yes **1**

No **5**  (**Go to 1.5**)

**1.2** How much time would you spend walking for travel on a typical day?

_______ Hours ________ minutes

##### 1.3 In a typical week, how many days do you walk for at least 10 minutes to get to and from places?

________ Days a week

**1.4** What is the intensity of walking?

Light (no change in breathing pattern) **1**

Moderate (make you breathe somewhat harder than normal) **2**

Vigorous (make you breathe much harder than normal) **3**

**1.5** Do you use a bicycle (pedal cycle) for at least 10 minutes continuously to get to and from places?

Yes **1**

No **5** (**Go to 2**)

**1.6** How much time would you spend bicycling for travel on a typical day?

________ Hours __________ minutes

**1.7** In a typical week how many days do you bicycle for at least 10 minutes to get to and from places?

__________ Days a Week

**1.8** What is the intensity of bicycling?

Light (no change in breathing pattern) **1**

Moderate (make you breathe somewhat harder than normal **2**

Vigorous (make you breathe much harder than normal **3**

**2. Leisure Time Activity**

I would like you to think about the things that you do in your free time.

**2.1** On average, how many hours per day do you spend doing activities in your free time where you are sitting down?

Weekdays: ____________ hours (to the nearest half hour)

Weekends: ____________ hours (to the nearest half hour)

**2.2** How often do you use stairs when an elevator is available?

(**please read out the options**)

Often **1**

Not very often **2**

Seldom **3**

Never **4**

**2.3** Which of the following do you do in your spare time (outside working hours)?

For each activity, if you do this at least once a week, then record the number of times **per week** that you do this activity. If you do this less than a week but at least once a month, record the number of times **per month** that you do this activity. Many of these activities will not be relevant to you. Only list those that you do at least once a month. If you do this **less than** **once a month**, then **do not** record it.

When estimating the duration of the activities, do not include rest periods when you are active.

|  |  | **How many times per week** | **How many times per month** | **Duration of each activity in minutes**  **each time** |
| --- | --- | --- | --- | --- |
| **Walking and Miscellaneous** | | | | |
| Walking for pleasure or exercise (e.g. walking with children or pets-do not include walking to get from one place to another) |  |  |  |  |
| Bicycling for pleasure |  |  |  |  |
| Dancing- ballroom, square, line and /or disco |  |  |  |  |
| Dancing- aerobic, ballet |  |  |  |  |
| Cross country hiking |  |  |  |  |
| Back packing (walking with a back pack) |  |  |  |  |
| Mountain climbing |  |  |  |  |
| Horseback riding |  |  |  |  |
| **Conditioning Exercise** | | | | |
| Home exercise (e.g. sit- ups, push-ups) |  |  |  |  |
| Health club exercise classes (e.g. aerobics) |  |  |  |  |
| Jog/ walk combinations |  |  |  |  |
| Balance exercises: Taiqi,  Qigong, breathing exercises |  |  |  |  |
| Running |  |  |  |  |
| Weight lifting |  |  |  |  |
| **Water Activities** | | | | |
| Water skiing |  |  |  |  |
| Sailing for pleasure |  |  |  |  |
| Sailing in competition |  |  |  |  |
| Canoeing or rowing for pleasure |  |  |  |  |
| Canoeing or rowing for competition |  |  |  |  |
| Swimming (at least 50 m in a pool) |  |  |  |  |
| Swimming at the beach |  |  |  |  |
| Scuba diving |  |  |  |  |
| Snorkeling |  |  |  |  |
|  |  |  |  |  |
|  | | **How many times per week** | **How many times per month** | **Duration of each activity in minutes**  **each time** |
| **Sports Activities** | | | | |
| Bowling |  |  |  |  |
| Volleyball |  |  |  |  |
| Table tennis |  |  |  |  |
| Tennis- singles |  |  |  |  |
| Tennis- doubles |  |  |  |  |
| Sepak Takraw |  |  |  |  |
| Martial arts- TKD, karate, judo, silat, lion dance, aikido |  |  |  |  |
| Softball |  |  |  |  |
| Badminton |  |  |  |  |
| Basketball/ netball- non game i.e. not keeping score |  |  |  |  |
| Basketball/ netball- game play (keeping score) |  |  |  |  |
| Basketball/ netball- refereeing |  |  |  |  |
| Rugby or American football |  |  |  |  |
| Soccer (football) |  |  |  |  |
| Squash |  |  |  |  |
| Paddle ball |  |  |  |  |
| Racket ball |  |  |  |  |
| Handball |  |  |  |  |
| Golf | Riding a powerkart/ buggy |  |  |  |
|  | Walking: pulling clubs on cart |  |  |  |
|  | Walking and carrying clubs |  |  |  |
| Fishing and hunting | Fishing in stream with wading boots |  |  |  |
|  | Hunting pheasant or grouse |  |  |  |
|  | Hunting rabbits, prairie chickens, squirrels, raccoons |  |  |  |
|  | Hunting larger game: deer, elk, bear, caribou |  |  |  |
|  |  |  |  |  |
| **Please list any other leisure time activities that you do regularly that have not been included in the list.** | | | | |
| **Others** |  |  |  |  |
|  |  |  |  |  |
|  |  |  |  |  |

**3. Occupational Physical Activity**

**3.1** Have you had a job (for which you have been paid or earned money) for more than 1 month in the last 3 months. This does not include work (e.g. housework) that you do in your own time. These will be covered in another section of the questionnaire. (**please circle the appropriate response**)

##### Yes 1

**No 5** (**Go to** **4**)

I would like to you to think about the time that you spend at work over the last 3 months. For each job that you’ve held in the last 3 months, I would like you to think about the activities that you do for that job. If you have only held 1 job in the last 3 months, then only 1 row should be filled in.

The total number of hours of activity (sitting, light, moderate, and heavy) should be added up to the hours worked per day.

| **No** | **Job Name** | **Working Hours Per Day** | **Days of work**  **Per week** | **Number of weeks in the last 3 months at the job** | **Hours spent sitting per day while work** | **Number of hours spent per day in each categories below when you are not sitting** | | |
| --- | --- | --- | --- | --- | --- | --- | --- | --- |
|  | | | | | | **Light activity** | **Moderate activity** | **Vigorous activity** |
|  |  |  |  |  |  |  |  |  |
|  |  |  |  |  |  |  |  |  |
|  |  |  |  |  |  |  |  |  |
|  |  |  |  |  |  |  |  |  |
|  |  |  |  |  |  |  |  |  |
|  |  |  |  |  |  |  |  |  |

##### Definitions

| **Intensity of activity** | **Examples** |
| --- | --- |
| **Light** | Standing still without heavy lifting |
|  | Light cleaning-ironing, cooking, washing, or dusting |
|  | Driving a car, bus, taxi, tractor |
|  | Jewelry making/ weaving |
|  | General office work |
|  | Occasional short distance walking |
|  |  |
| Moderate | Carrying light loads |
|  | Continuous walking |
|  | Heavy cleaning- mopping, sweeping, scrubbing, vacuuming |
|  | Gardening- planting or weeding |
|  | Painting/ plastering |
| Heavy | Carrying moderate to heavy loads |
|  | Heavy construction |
|  | Farming- hoeing, digging, mowing, raking |
|  | Digging, ditches/ shoveling |
|  | Tree-pole climbing |
|  | Chopping or sawing wood |
|  | Water/ coal or wood hauling |

**4. Household Activity**

Now I would like you to think about the activities that you perform in order to look after your own home. Please list the amount of time that you spend on the following activities.

| Activity | **Hours per day** | **Days per week** |
| --- | --- | --- |
| Shopping (e.g. groceries, clothes): excluding the time to get there |  |  |
| Stair climbing while carrying the load |  |  |
| Laundry (time loading, unloading, hanging, or folding only) |  |  |
| Light housework; tidying/ dusting, sweeping, collecting trash in the home, polishing, indoor gardening, ironing |  |  |
| Heavy housework: vacuuming, mopping, scrubbing floors and walls, moving furniture, boxes and garbage cans. |  |  |
|  |  |  |
| Food preparation: (10 +minutes in duration): chopping, stirring, moving about to get food items/ pans etc. |  |  |
| Food service (10+ minutes duration): setting table, carrying, food, serving food. |  |  |
|  |  |  |
| Dish washing (10+ minutes in duration): clearing table, washing/ drying dishes, putting dishes away. |  |  |
| Light home repair: small appliances repair, light home maintenance/ repair. |  |  |
| Heavy home repair: painting, carpentry, washing/ polishing car |  |  |
| Others: |  |  |
|  |  |  |
| Yard work |  |  |
| Gardening: planting, weeding, digging, or hoeing |  |  |
| Lawn mowing (walking only) |  |  |
| Clearing walks, driveways: sweeping, shoveling, raking |  |  |
|  |  |  |
| Looking after elderly persons or children |  |  |
| Older or disabled person (lifting, pushing wheelchair) |  |  |
| Childcare (lifting, carrying or pushing stroller) |  |  |
