# Supplementary material for: Validity of the international physical activity questionnaire and the Singapore prospective study program physical activity questionnaire in a multiethnic urban Asian population
Source: BMC Med Res Methodol. 2011 Oct 13;11:141. doi: 10.1186/1471-2288-11-141 (PMC3212806; doi:10.1186/1471-2288-11-141)
Supplement: Additional file 2 — "Correlation between IPAQ and SP2PAQ measurements of energy expenditure from physical activity" for spearman correlations between IPAQ and SP2PAQ measurements of energy expenditure from moderate and vigorous activity. [file 1471-2288-11-141-S2.DOC]

| **Additional file 2. Correlation between IPAQ and SP2PAQ measurements of energy expenditure from physical activity** | | |
| --- | --- | --- |
|  | **Moderate activity** | **Vigorous activity** |
|  | **Correlation** | **Correlation** |
|  | 0.55** | 0.27** |
| **Stratified by age group** |  |  |
| ≤40 years(N=87) | 0.43** | 0.44* |
| >40 years(N=65) | 0.58** | 0.004 |
| **Stratified by gender** |  |  |
| Male(N=64) | 0.57** | 0.30* |
| Female(N=88) | 0.55** | 0.19 |
| **Stratified by Ethnicity** |  |  |
| Chinese(N=66) | 0.53** | 0.37* |
| Malay(N=34) | 0.51* | 0.17 |
| Indian(N=52) | 0.49* | 0.24 |

*p value<0.05, ** p value<0.0001

IPAQ= International Physical Activity Questionnaire; SP2PAQ= Singapore Prospective Study Program Physical Activity Questionnaire
